# Plasmid replication initiator protein TrfA represses the host type III secretion system in *Pseudomonas aeruginosa*

**DOI:** 10.1128/mbio.02784-25

**Published:** 2025-11-05

**Authors:** Yu Zhang, Liwen Yin, Qi Liu, Lepeng Wang, Chenyu Shen, Yifan Bian, Zhenzhen Ma, Un-Hwan Ha, Zhihui Cheng, Weihui Wu, Shouguang Jin, Yongxin Jin

**Affiliations:** 1 State Key Laboratory of Medicinal Chemical Biology, Key Laboratory of Molecular Microbiology and Technology of the Ministry of Education, Department of Microbiology, College of Life Sciences, Nankai University 616162, Tianjin, China; 2 Department of Biotechnology and Bioinformatics, Korea University 65692 , Sejong, Republic of Korea; University of Nebraska Medical Center, Omaha, Nebraska, USA

**Keywords:** *P. aeruginosa*, type III secretion system, TrfA, ExsA

## Abstract

*Pseudomonas aeruginosa* is an environmental bacterium and opportunistic human pathogen capable of causing a wide range of infections in humans. The type III secretion system (T3SS) plays a key role in the pathogenesis of acute *P. aeruginosa* infections. Expression of the T3SS is controlled by a master activator ExsA, whose transcription is driven by its operon promoter P*_exsC_* as well as its own promoter P*_exsA_*. Here, we found that plasmid replication initiation protein TrfA acts as a repressor for the T3SS gene expression in *P. aeruginosa*. Production of the TrfA reduced the expression of the T3SS genes and the bacterial pathogenicity in a mouse acute pneumonia model. Further analysis revealed that the expression of *trfA* resulted in upregulation of the novel gene PA5530, which mediates reduced cAMP production and decreased the expression of T3SS in PAK. In addition, we demonstrate that TrfA regulates the T3SS by directly binding to and modulating the P*_exsC_* promoter. Of note, the clinical plasmid harboring the *trfA* gene also leads to downregulation of T3SS in *P. aeruginosa*. Overall, the results from this study reveal the effect of the plasmid replication initiation protein on the expression of its host chromosomal genes.

**IMPORTANCE:**

As extrachromosomal elements, plasmids are well known for their role in conferring advantageous attributes to hosts, including antibiotic resistance, heavy metal resistance, the ability to degrade xenobiotics, and osmotolerance. Although several host chromosomally encoded proteins are required for the replication and maintenance of plasmids in their hosts, few proteins from plasmids have been reported to affect the host chromosomal gene expression. In this study, we identified TrfA, a plasmid replication initiation protein, as a novel repressor of the T3SS in *P. aeruginosa*. We also elucidated the regulatory mechanism of T3SS repression mediated by the TrfA. The significance of this work is in the identification of the replication initiation protein of a naturally occurring plasmid from *P. aeruginosa* functioning as a regulator of the expression of chromosomal genes in its host *P. aeruginosa*.

## INTRODUCTION

*Pseudomonas aeruginosa* is an environmental bacterium and opportunistic human pathogen that is responsible for varieties of infections in patients with immune compromise or cystic fibrosis patients (1). The successful infection of *P. aeruginosa* is largely due to its ability to produce a wide range of virulence factors, including type III secretion system (T3SS), rhamnolipids, pyocyanin, biofilm, and motilities (1–3). Among them, the T3SS plays a key role in the pathogenesis of acute *P. aeruginosa* infections (4, 5). It directly injects toxin proteins into the cytosol of host cells, resulting in cell death or malfunction (5, 6). Expression of all T3SS genes is activated by the master regulator ExsA (7). ExsA is an AraC/XylS family transcriptional regulator that binds to the promoters of T3SS to activate their expression, including *exsA* itself (8). Transcription of *exsA* is driven by its operon promoter P*_exsC_* as well as its own promoter P*_exsA_*, which are modulated by ExsA and Vfr-cAMP, respectively (7, 9). Many other regulators control the expression of T3SS via modulating *exsA* transcription, translation, or its regulator activity in *P. aeruginosa* (7, 10).

Cyclic adenosine monophosphate (cAMP) is a secondary messenger that functions as a cofactor for the transcriptional regulator Vfr (virulence factor regulator) to bind to and regulate the expressions of multiple genes (11). In *P. aeruginosa*, cAMP-Vfr regulates the expressions of over 200 genes, including those associated with T3SS, type IV pili, flagellar, and quorum sensing systems (12, 13). Intracellular cAMP levels are tightly controlled by its synthesis from ATP and degradation into AMP. Two adenylate cyclases, CyaA and CyaB, have been found to synthesize cAMP in *P. aeruginosa*, with CyaB playing a major role in cAMP synthesis (12). The phosphodiesterase CpdA has been described to be responsible for cAMP degradation in *P. aeruginosa* (14).

RK2, a self-transmissible plasmid belonging to the IncP1 incompatibility group, was originally isolated from *P. aeruginosa* and found to have an extraordinarily broad host range, capable of replication and maintenance in nearly all gram-negative bacteria (15, 16). The replication initiation protein TrfA (trans-acting factor A) and the replication origin sequence *oriV* are sufficient for plasmid RK2 replication in a broad range of hosts (17). Host proteins are also required for RK2 replication, including DnaA, DnaB, DnaC, DnaG primase, DNA gyrase, HU, SSB, and Pol III holoenzyme (18). Plasmids are well known for their roles in conferring host resistance to various agents or novel phenotypes. However, whether and how the plasmid affects the host chromosomal gene expression remains largely unknown.

In this study, we found that the plasmid replication initiation protein TrfA represses the expression of the T3SS in *P. aeruginosa*. We show that the expression of *trfA* in *P. aeruginosa* reduced the expression of T3SS genes and pathogenicity in a mouse acute pneumonia model. We demonstrate that TrfA controls the T3SS by directly binding to and modulating the *exsCEBA* operon promoter P*_exsC_* as well as through upregulation of PA5530, a gene encoding a C5-dicarboxylate transporter. In addition, *trfA* expression also decreased intracellular cAMP levels and altered cAMP-related phenotypes in *P. aeruginosa*. The present work reveals the effect of a plasmid-encoded replication initiation protein on the expression of its host chromosomal genes in *P. aeruginosa*.

## RESULTS

### TrfA represses the expression of ExoS in *P. aeruginosa*

**Fig 1:**
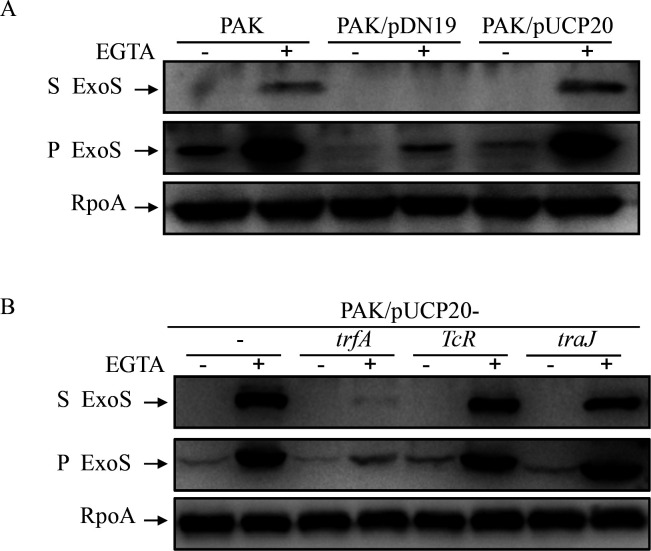
TrfA represses ExoS expression and secretion in the PAK strain. (**A and B**) Expression and secretion of ExoS in the indicated strains. Bacterial cells were grown to an OD_600_ of 1.0 in LB with 0 (−) or 5 mM (+) EGTA. Proteins in supernatants (S) and pellets (P) from equivalent bacterial cells were separated by 12% SDS-PAGE gels and probed with anti-ExoS antibody or anti-RpoA antibody. (**B**) TrfA, TcR, and TraJ encoding genes from pDN19 were cloned into pUCP20 and expressed in PAK strains.

 During our studies of T3SS in *P. aeruginosa*, we observed that secretion and expression of the ExoS were significantly decreased in PAK when harboring a shuttle vector pDN19 compared to that containing another shuttle vector pUCP20 or PAK alone under both T3SS-inducing and noninducing conditions (Fig. 1A). The pDN19 plasmid contains three protein-encoding genes, *TcR* (*tetA*), encoding a 42.2 kD tetracycline efflux protein that confers tetracycline resistance; *trfA*, encoding a 44 kD trans-acting replication protein that binds to and activates *oriV* of pDN19; and *traJ*, which encodes a 13.5 kD protein that recognizes the *oriT* sequence on the pDN19. The reduced expression and secretion of ExoS in the PAK/pDN19 strain might be caused by the proteins encoded on the pDN19. To determine if the three genes are responsible for the decreased ExoS expression in PAK, each gene was cloned into pUCP20 and introduced into the PAK strain. Western blot assay clearly showed that the *trfA* gene could suppress both expression and secretion of ExoS in PAK, while *TcR* and *traJ* had no obvious effect on the ExoS compared to PAK containing the empty vector pUCP20 (Fig. 1B). As expected, the expression of TcR conferred PAK a 32-fold increase in the MIC against tetracycline (Fig. S1).

### TrfA represses the T3SS and reduces bacterial pathogenicity

**Fig 2:**
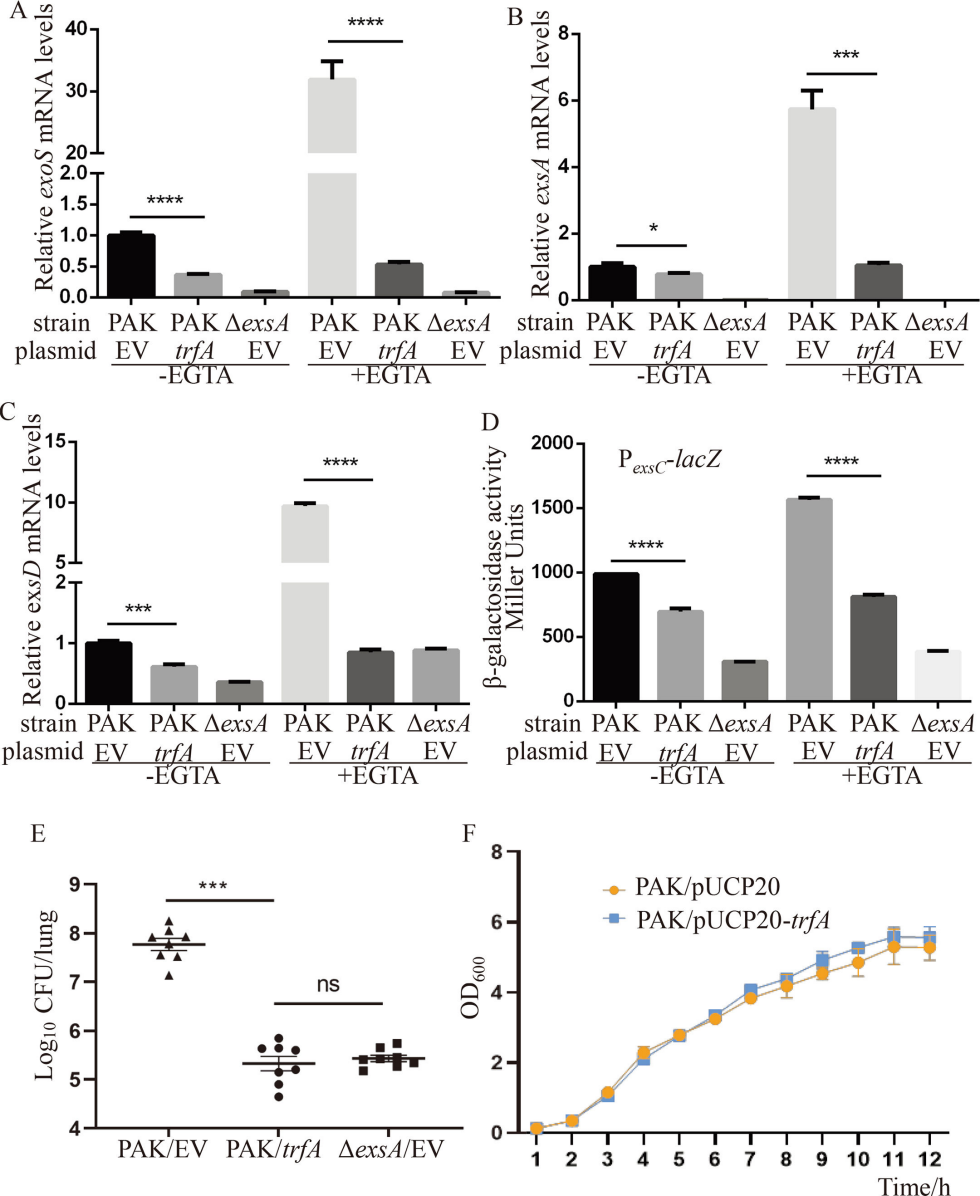
TrfA represses the T3SS and reduces bacterial pathogenicity. (**A–C**) Relative mRNA levels of *exoS*, *exsA*, and *exsD* in PAK/pUCP20, PAK/pUCP20-*trfA*, and PAKΔ*exsA*/pUCP20 strains. Total RNA was isolated under T3SS noninducing and inducing conditions, and the relative mRNA levels were determined by real-time qPCR using *rpsL* as the internal control. *P < 0.05, ***P < 0.001, and ****P < 0.0001, by Student’s *t* test. (**D**) PAK/pUCP20, PAK/pUCP20-*trfA,* and PAKΔ*exsA*/pUCP20 containing the P*_exsC_-lacZ* transcriptional reporter plasmid were grown to an OD_600_ of 1.0 in LB with 0 (−) or 5 mM (+) EGTA and subjected to β-galactosidase assays. Each assay was performed in triplicate, and the error bars indicate standard deviations. ****P < 0.0001 compared with PAK/pUCP20 by Student’s *t* test. (**E**) Mice were intranasally inoculated with 2 × 10^7^ CFU bacteria of PAK/pUCP20, PAK/pUCP20-*trfA,* and PAKΔ*exsA*/pUCP20. After 12 hours, the mice were sacrificed, and the lungs were isolated and homogenized. The bacterial loads were determined by serial dilution and plating. The central bar represents the mean, and the error bar indicates the standard error. ns, not significant, ***, *P* < 0.001 by the Mann-Whitney test. (**F**) Growth curve of PAK/pUCP20 and PAK/pUCP20-*trfA* in the LB medium.

To determine whether the repression of T3SS expression mediated by TrfA is limited to the effector protein ExoS or occurs on the whole type III secretion apparatus, we compared the relative mRNA levels of *exsA*, the gene encoding the T3SS master transcriptional activator ExsA, in PAK with or without TrfA by real-time qPCR. As shown in Fig. 2, similar to *exoS*, *exsA* and another regulator gene, *exsD,* were significantly downregulated in PAK/pUCP20-*trfA* under both T3SS inducing and non-inducing conditions. To further confirm the decreased transcription of T3SS, a P*_exsC_-lacZ* reporter plasmid for the transcription of the *exsCEBA* operon was introduced into PAK/pUCP20 and PAK/pUCP20-*trfA* strains (19). β-galactosidase activity measurements showed that transcription of P*_exsC_* was significantly decreased in PAK/pUCP20-*trfA* under both T3SS-inducing and noninducing conditions, although not to the levels of that in the PAKΔ*exsA*/pUCP20 strain (Fig. 2D). These results demonstrate a repressive role of TrfA in the expression of the T3SS in *P. aeruginosa*.

Since the T3SS plays a critical role in acute infections of *P. aeruginosa* (20), the functional connection between TrfA and the T3SS prompted us to determine the role of TrfA in the pathogenesis of a mouse acute pneumonia model. Accordingly, female BALB/c mice (6 to 8 weeks old) were intranasally infected with 2 × 10^7^ CFU of PAK/pUCP20 or PAK/pUCP20-*trfA*. Twelve hours after infection, murine lungs were isolated, homogenized, and plated on LB agar plates to determine the bacterial loads. Compared to the PAK/pUCP20 strain, the bacterial number of PAK/pUCP20-*trfA* recovered from the lungs was significantly lower, even as low as that of a T3SS-defective PAKΔ*exsA*/pUCP20 strain (Fig. 2E), indicating reduced pathogenicity. When grown in the LB medium, the PAK/pUCP20-*trfA* displayed an indistinguishable growth rate from that of PAK/pUCP20 (Fig. 2F).

To further examine whether TrfA represses the expression of the T3SS in other *P. aeruginosa* strains, we introduced pUCP20-*trfA* into two other lab strains PAO1 and PA14. Western blot assay and real-time qPCR showed that the expression of *trfA* represses the T3SS in both PAO1 and PA14 strains (Fig. S2).

### PA5530 is involved in the TrfA-mediated repression of the T3SS

**Fig 3:**
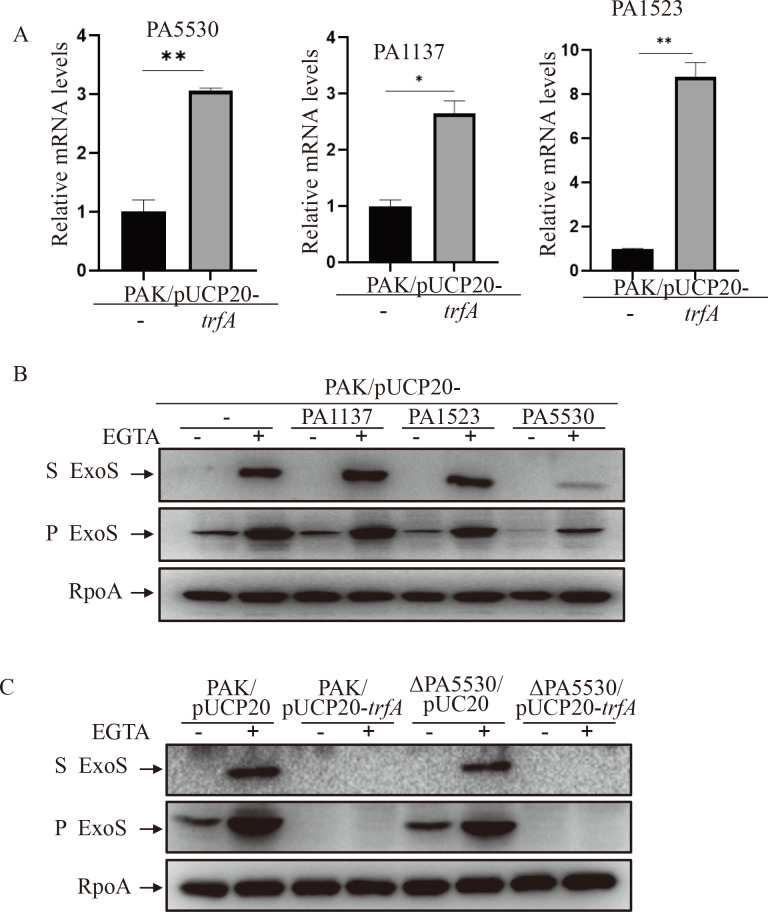
PA5530 is involved in the TrfA-mediated repression of the T3SS. (**A**) Relative mRNA levels of PA5530, PA1137, and PA1523 in the PAK/pUCP20 and PAK/pUCP20-*trfA* strains. Total RNA was isolated under T3SS noninducing conditions, and the relative mRNA levels were determined by real-time qPCR using *rpsL* as the internal control. *P < 0.05, **P < 0.01, by Student’s *t* test. (**B and C**) Expression and secretion of ExoS in the indicated strains. Bacterial cells were grown to an OD_600_ of 1.0 in LB with 0 (−) or 5 mM (+) EGTA. Proteins in supernatants (S) and pellets (P) from equivalent bacterial cells were separated by 12% SDS-PAGE gels and probed with anti-ExoS antibody or anti-RpoA antibody.

To elucidate the regulatory mechanism of T3SS repression by TrfA, we compared the global gene transcription profiles between strains PAK/pUCP20 and PAK/pUCP20-*trfA*. Under T3SS non-inducing conditions, a total of 23 genes were significantly altered between the two strains (Table S1). Among them, 11 genes belonging to the T3SS were decreased in the PAK/pUCP20-*trfA* strain (Table 1), which is consistent with the downregulation of the T3SS by TrfA. Interestingly, only three genes were upregulated when *trfA* was expressed in the PAK, including PA1137, PA1523, and PA5530 (Table S1). To confirm the upregulation of these genes in the PAK/pUCP20-*trfA* strain, we examined their relative mRNA levels using a real-time qPCR assay. As expected, the relative mRNA levels of PA1137, PA1523, and PA5530 were much higher in PAK/pUCP20-*trfA* than in PAK/pUCP20 (Fig. 3A). According to the description on the *Pseudomonas* website (www.pseudomonas.com), PA1137 encodes a probable oxidoreductase, PA1523 encodes a xanthine dehydrogenase, and PA5530 encodes a C5-dicarboxylate transporter. To examine whether any of the three upregulated genes was involved in the repression of the T3SS by TrfA, each of them was cloned into pUCP20 and introduced into PAK. Western blot analysis clearly showed that PA5530 suppressed the expression of ExoS under both T3SS-inducing and noninducing conditions, while PA1137 and PA1523 had no detectable effect on the ExoS expression (Fig. 3B). These results suggested that PA5530 is involved in the suppression of T3SS by TrfA.

**TABLE 1:** mRNA levels of genes related to T3SS in *PAK/pUCP20-trfA* compared to those in PAK/pUCP20

Gene ID	Gene name	Gene function	Fold change (_PAK-TrfA/PAK-pUCP20_)	*P* value
PA1691	*pscT*	Translocation protein in type III secretion	0.04	8.01E-05
PA1693	*pscR*	TTranslocation protein in type III secretion	0.04	6.95E-05
PA1694	*pscQ*	Translocation protein in type III secretion	0.04	7.62E-05
PA1695	*pscP*	Translocation protein in type III secretion	0.04	4.47E-05
PA1697	*pscN*	ATP synthase in type III secretion system	0.04	5.91E-05
PA1698	*popN*	Type III secretion outer membrane protein PopN precursor	0.04	0.000119
PA1699	*pcr1*	Pcr1	0.05	0.000175
PA1703	*pcrD*	Type III secretory apparatus protein PcrD	0.02	1.88E-05
PA1708	*popB*	Translocator protein PopB	0.04	0.000135
PA1709	*popD*	Translocator outer membrane protein PopD precursor	0.04	7.20E-05
PA1722	*pscI*	Type III export protein PscI	0.04	0.000127

To further validate its repressive function, the PA5530 gene was deleted in the PAK strain, where the T3SS was expected to increase. Unexpectedly, however, the expression and secretion of ExoS did not increase in the resulting strain PAKΔPA5530 compared to that in the wild-type strain PAK (Fig. 3C). Additionally, the expression of *trfA* in PAKΔPA5530 resulted in a similar decrease in the expression of ExoS as in PAK (Fig. 3C). Therefore, these data suggested that PA5530 might function as a T3SS repressor only under certain conditions when it is upregulated, while under regular conditions, additional TrfA-mediated regulatory pathways may exist in *P. aeruginosa*.

### The cAMP level was decreased when *trfA* was expressed in *P. aeruginosa*

**Fig 4:**
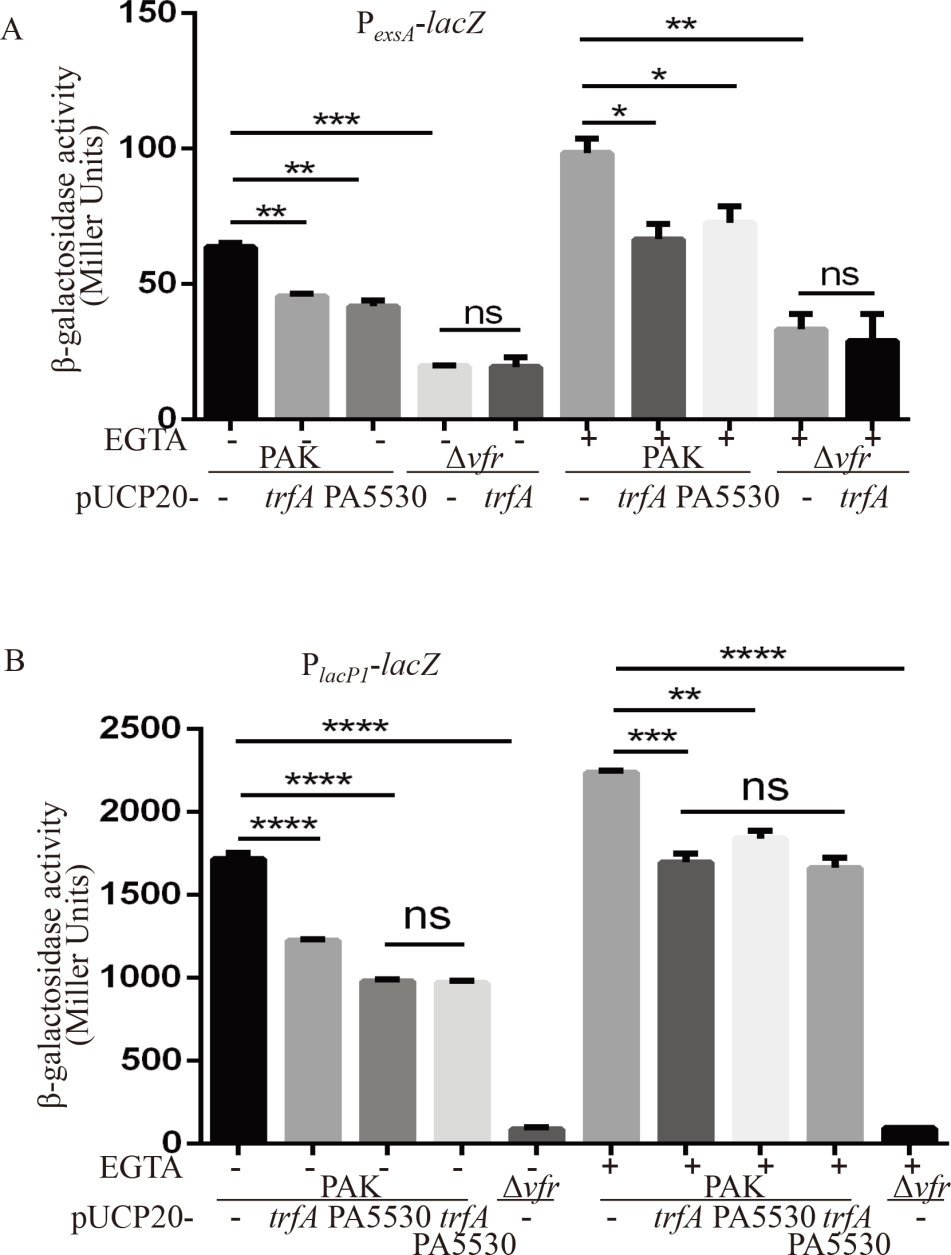
cAMP levels were decreased when *trfA* was expressed in PAK. β-galactosidase activity assay. The indicated strains containing the P*_exsA_-lacZ* (**A**) or P*_lacP1_-lacZ* (**B**) transcriptional reporter plasmid were grown to an OD_600_ of 1.0 in LB with 0 (−) or 5 mM (+) EGTA and subjected to β-galactosidase assays. Each assay was performed in triplicate, and the error bars indicate standard deviations. ns, not significant, *P < 0.05, **P < 0.01, ***P < 0.001, and ****P < 0.0001, by Student’s *t* test.

All of the T3SS genes are directly controlled by ExsA (4, 7), and we found that the transcriptional activity of P*_exsC_* was lower in the PAK/pUCP20-*trfA* strain (Fig. 2D). Since several regulators have been found to regulate the T3SS by directly modulating the *exsA* gene own promoter, we examined the effect of TrfA on the P*_exsA_* transcriptional activity using a P*_exsA_-lacZ* transcriptional reporter construct (19). Vfr has been shown to directly activate *exsA* transcription from the P*_exsA_* promoter (9); therefore, PAKΔ*vfr* was used as a control. As expected, the PAKΔ*vfr* displayed a significantly decreased β-galactosidase activity compared to the PAK strain (Fig. 4A). The β-galactosidase levels in PAK/pUCP20-*trfA* background were significantly lower than those in PAK/pUCP20 under both T3SS-inducing and noninducing conditions (Fig. 4A), while the β-galactosidase levels of P_*tac*_-*lacZ* were similar between PAK/pUCP20 and PAK/pUCP20-*trfA* (Fig. S3A). As the cAMP-Vfr system directly activates *exsA* transcription from P*_exsA_* (9) and the transcriptional level of *vfr* was not affected by TrfA in PAK according to the RNAseq data, we further compared the cAMP levels between PAK/pUCP20 and PAK/pUCP20-*trfA* using a P*_lacP1_-lacZ* reporter construct, which has been used widely as an indicator for intracellular cAMP levels (21, 22). As shown in Fig. 4B, β-galactosidase activity of P*_lacP1_* was significantly decreased in PAKΔ*vfr*, which is consistent with previous studies (21, 22). Of note, in the background of PAK/pUCP20-*trfA*, the β-galactosidase activity was much lower than that in PAK/pUCP20 under both T3SS-inducing and noninducing conditions, indicating decreased intracellular cAMP levels in PAK/pUCP20-*trfA* (Fig. 4B). The above data indicated that lower cAMP levels might contribute to the decreased P*_exsA_* promoter activity in PAK/pUCP20-*trfA*. In agreement with this, the *trfA* expression did not further decrease the P*_exsA_* promoter activity in PAKΔ*vfr* under either T3SS-inducing or noninducing conditions (Fig. 4A).

Since PA5530 is involved in the TrfA-mediated repression of the T3SS in PAK, we wanted to determine whether the expression of PA5530 could decrease the intracellular cAMP levels and influence the P*_exsA_* activity in PAK, similar to that of TrfA. To test this possibility, the P*_lacP1_-lacZ* or P*_exsA_-lacZ* reporter construct was introduced into PAK/pUCP20-PA5530, and β-galactosidase activity was measured under T3SS-inducing and noninducing conditions. As shown in Fig. 4, similar to that in PAK/pUCP20-*trfA*, the β-galactosidase activities of both P*_lacP1_-lacZ* and P*_exsA_-lacZ* were significantly decreased in the PAK/pUCP20-PA5530 strain background compared to those in the PAK/pUCP20 background. Since individual expressions of *trfA* and PA5530 decreased cAMP levels in PAK, we want to know whether production of both proteins has an additive effect on cAMP levels. *trfA* and PA5530 were co-expressed with the pUCP20 plasmid in PAK containing the P*_lacP1_-lacZ*. As shown in Fig. 4B, the co-production of TrfA and PA5530 did not further decrease the β-galactosidase activity, indicating that TrfA and PA5530 are functioning on the same regulatory pathway.

### Expression of *cyaB* decreased when TrfA was overexpressed in PAK

**Fig 5:**
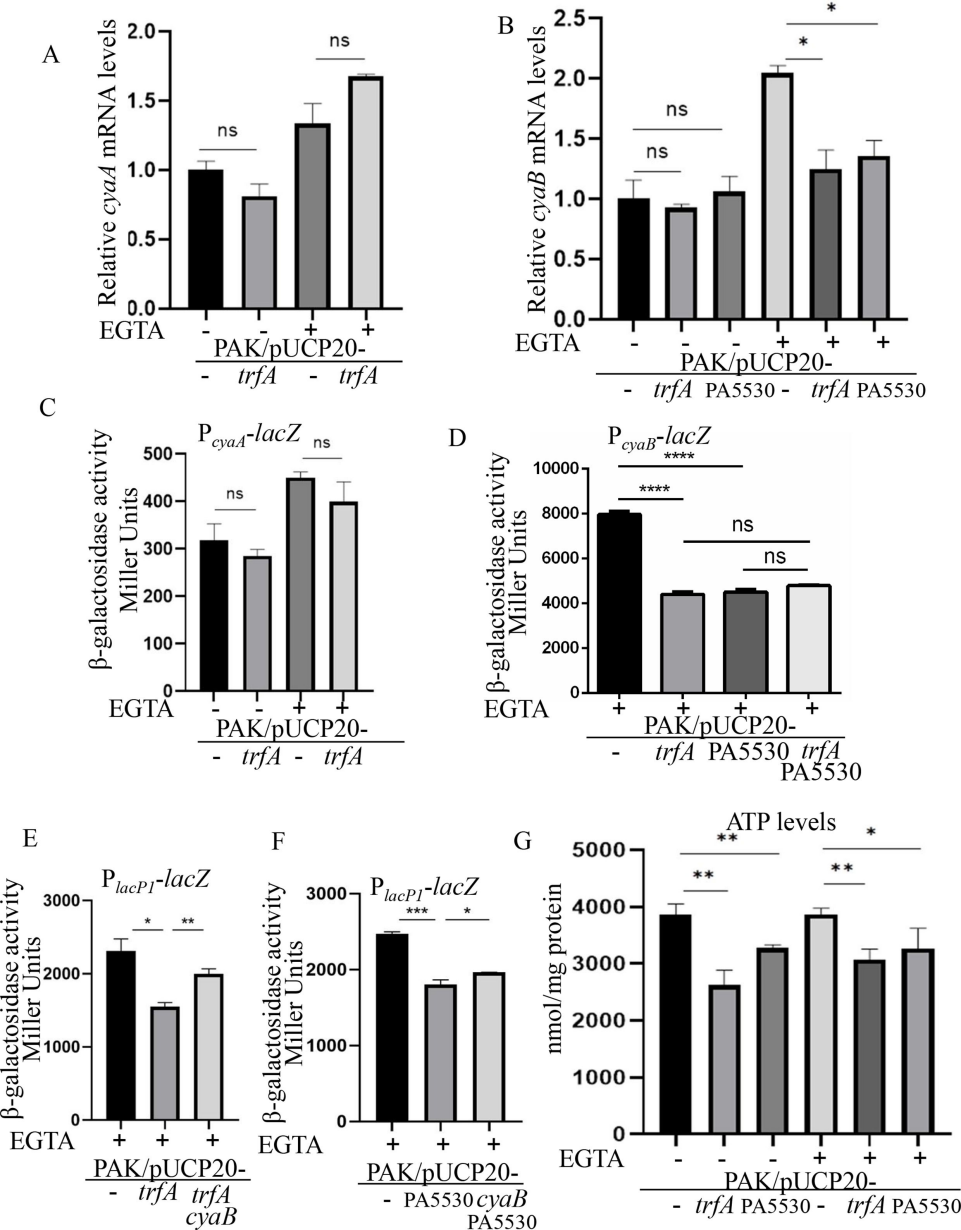
Expression levels of *cyaA* and *cyaB*, as well as ATP levels in the indicated strains. (**A and B**) Relative mRNA levels of *cyaA* and *cyaB* in the PAK/pUCP20 and PAK/pUCP20-*trfA* or PAK/pUCP20-PA5530 strains. Total RNA was isolated under T3SS inducing and noninducing conditions, and the relative mRNA levels were determined by real-time qPCR using *rpsL* as the internal control. (**C–F**) β-galactosidase activity assay. Indicated bacterial strains containing the P*_cyaA_-lacZ* (**C**), P*_cyaB_-lacZ* (**D**), or P*_lacP1_-lacZ* (**E and F**) transcriptional reporter plasmid were grown to an OD_600_ of 1.0 in LB with 0 (−) or 5 mM (+) EGTA and subjected to β-galactosidase assays. Each assay was performed in triplicate, and the error bars indicate standard deviations. (**G**) ATP levels in the indicated strains. ns, not significant, *P < 0.05, **P < 0.01, ***P < 0.001, and ****P < 0.0001, by Student’s *t* test.

In *P. aeruginosa*, cAMP is synthesized by the adenylate cyclases CyaA and CyaB (12, 23). To understand the molecular mechanism of decreased cAMP in PAK/pUCP20-*trfA*, we determined the relative mRNA levels of *cyaA* and *cyaB* by real-time qPCR. Consistent with the transcriptional profile analysis, the mRNA levels of *cyaA* were not altered by the expression of *trfA* in PAK (Fig. 5A). Consistent with a previous study (12), *cyaB* was upregulated under the low-calcium condition (Fig. 5B). Of note, the *cyaB* gene displayed a significant decrease in PAK/pUCP20-*trfA* under T3SS inducing condition (Fig. 5B). To confirm this observation, we constructed and utilized two *lacZ* transcriptional reporter plasmids, P*_cyaA_-lacZ* and P*_cyaB_-lacZ*. In agreement with the real-time qPCR results, no obvious alteration in β-galactosidase activity was detected for P*_cyaA_-lacZ* between PAK/pUCP20 and PAK/pUCP20-*trfA* (Fig. 5C), while β-galactosidase activity for P*_cyaB_-lacZ* was significantly decreased in PAK/pUCP20-*trfA* under T3SS inducing condition (Fig. 5D). In addition, PAK/pUCP20-PA5530 displayed a similar expression pattern of *cyaB* as that in PAK/pUCP20-*trfA* (Fig. 5B and D). However, co-expression of *trfA* and PA5530 showed no additive effect on the expression of *cyaB* in PAK (Fig. 5D), which agrees with the non-additive effect on cAMP levels (Fig. 4B). If a decreased expression of *cyaB* resulted in the reduced intracellular level of cAMP, ectopic expression of the *cyaB* should increase the cAMP levels in PAK/pUCP20-*trfA* and PAK/pUCP20-PA5530. As shown in Fig. 5E and F, β-galactosidase activities for P*_lacP1_-lacZ* were significantly increased in PAK/pUCP20-*trfA* and PAK/pUCP20-PA5530 when the *cyaB* was ectopically expressed, although not to the levels seen in PAK/pUCP20. In combination, these results suggested that the decreased intracellular cAMP levels might be due to the altered expression of its major synthase CyaB.

### ATP level decreased when TrfA was overexpressed in PAK

Since cAMP is produced from adenosine triphosphate (ATP), decreased ATP availability in PAK/pUCP20-*trfA* or PAK/PA5530 might lead to reduced cAMP levels. Therefore, we further examined the intracellular ATP levels in PAK/pUCP20-*trfA* and PAK/pUCP20-PA5530 using an ATP assay kit (Beyotime Biotec, Shanghai, China). Indeed, the ATP levels were significantly lower in PAK/pUCP20-*trfA* and PAK/pUCP20-PA5530 than in PAK/pUCP20 (Fig. 5G).

### TrfA binds to the P*_exsC_* to repress the T3SS directly

**Fig 6:**
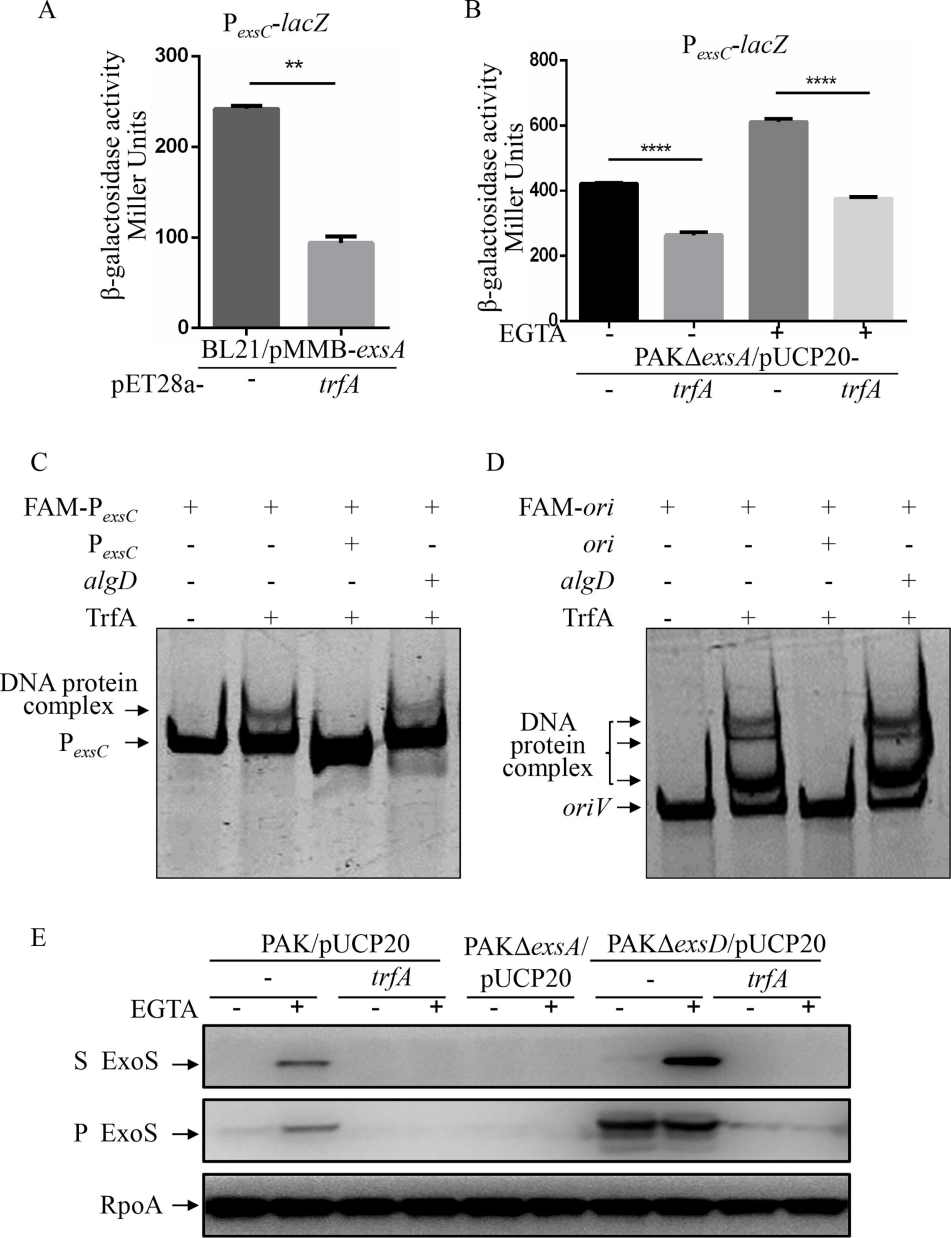
TrfA binds to the P*_exsC_* to repress the T3SS directly. (**A and B**) β-galactosidase activity assay. BL21/pET28a and BL21/pET28a-*trfA* containing the P*_exsC_-lacZ* and pMMB67EH-*exsA* plasmids (**A**), as well as PAKΔ*exsA*/P*_exsC_-lacZ* containing pUCP20 or pUCP20-*trfA* were grown to an OD_600_ of 1.0 in LB and subjected to β-galactosidase assays. Each assay was performed in triplicate, and the error bars indicate standard deviations. **P < 0.01, ****P < 0.0001, by Student’s *t* test. (**C and D**) TrfA binds to the *exsC* promoter, as well as the *oriV* of pDN19 plasmid. 6-FAM labeled DNA probe (10 ng) was incubated with 0 or 0.9 µM TrfA on ice for 30 min. 50-fold excess of unlabeled DNA was used as competitors. The shifted band is indicated by an arrowhead. (**E**) Expression and secretion of ExoS in the indicated strains. Bacterial cells were grown to an OD_600_ of 1.0 in LB with 0 (−) or 5 mM (+) EGTA. Proteins in supernatants (S) and pellets (P) from equivalent bacterial cells were separated by 12% SDS-PAGE gels and probed with anti-ExoS antibody or anti-RpoA antibody.

Based on the facts that TrfA is able to bind to and activate *oriV* of plasmids and that an additional TrfA-mediated T3SS regulatory pathway may exist in *P. aeruginosa*, we wanted to know whether TrfA can bind to and repress the P*_exsC_* directly. The *trfA* was cloned into pET28a and introduced into the *E. coli* BL21(DE3) containing the P*_exsC_-lacZ* and ExsA (transcriptional activators for P*_exsC_*). As the β-galactosidase activity assay result shown in Fig. 6A, compared to the empty vector pET28a, the expression of *trfA* significantly decreased the β-galactosidase activity in BL21 containing P*_exsC_-lacZ,* but not in the negative control P*_tac_-lacZ* (Fig. S3B). To further confirm the direct repression of the P*_exsC_* activity mediated by TrfA, we examined the P*_exsC_* promoter activity in the PAKΔ*exsA* strain. As shown in Fig. 6B, in the absence of *exsA*, the promoter activity of P*_exsC_* decreased in comparison with that seen in wild-type PAK (Fig. 2D). Importantly, in the absence of *exsA*, the expression of *trfA* still resulted in significant decreases in β-galactosidase activity under both T3SS inducing and noninducing conditions (Fig. 6B). These results above indicate direct repression of the P*_exsC_* promoter by TrfA specifically.

To further determine the direct binding of TrfA to the P*_exsC_* promoter, we performed EMSA using a DNA fragment corresponding to the promoter region of *exsC*. The C-terminal His-tagged TrfA was overexpressed in pET28a and purified from *E. coli* BL21(DE3). The DNA fragments corresponding to the *oriV* of pDN19 plasmid were used as a positive control. The FAM-labeled DNA fragment derived from the *exsC* promoter region shifted upon incubation with TrfA-His (Fig. 6C and D). The binding specificity was validated by a detectable competitive binding by its unlabeled DNA, but not by an unrelated DNA fragment corresponding to the upstream of the *algD* gene (Fig. 6C and D). These results indicated that TrfA directly binds to the *exsCEBA* operon promoter *P_exsC_*.

Since the TrfA functions as a repressor via directly binding to the promoter of *exsCEBA*, the expression of *trfA* would also inhibit the T3SS when the ExsA is not sequestered by ExsD and fully active. Therefore, we examined the expression and secretion of ExoS in the PAKΔ*exsD*/pUCP20-*trfA* strain. As shown in Fig. 6E, expression and secretion of ExoS were increased in PAKΔ*exsD* compared to the parental PAK strain. However, the expression of the *trfA* reduced the expression and secretion of ExoS in PAKΔ*exsD*. Consistent with this, the P*_exsC_* activity was increased in PAKΔ*exsD*/pUCP20 compared to PAK/pUCP20, while the TrfA significantly reduced the P*_exsC_* activity in PAKΔ*exsD* (Fig. S3C).

### Global regulatory role of the TrfA in *P. aeruginosa*

**Fig 7:**
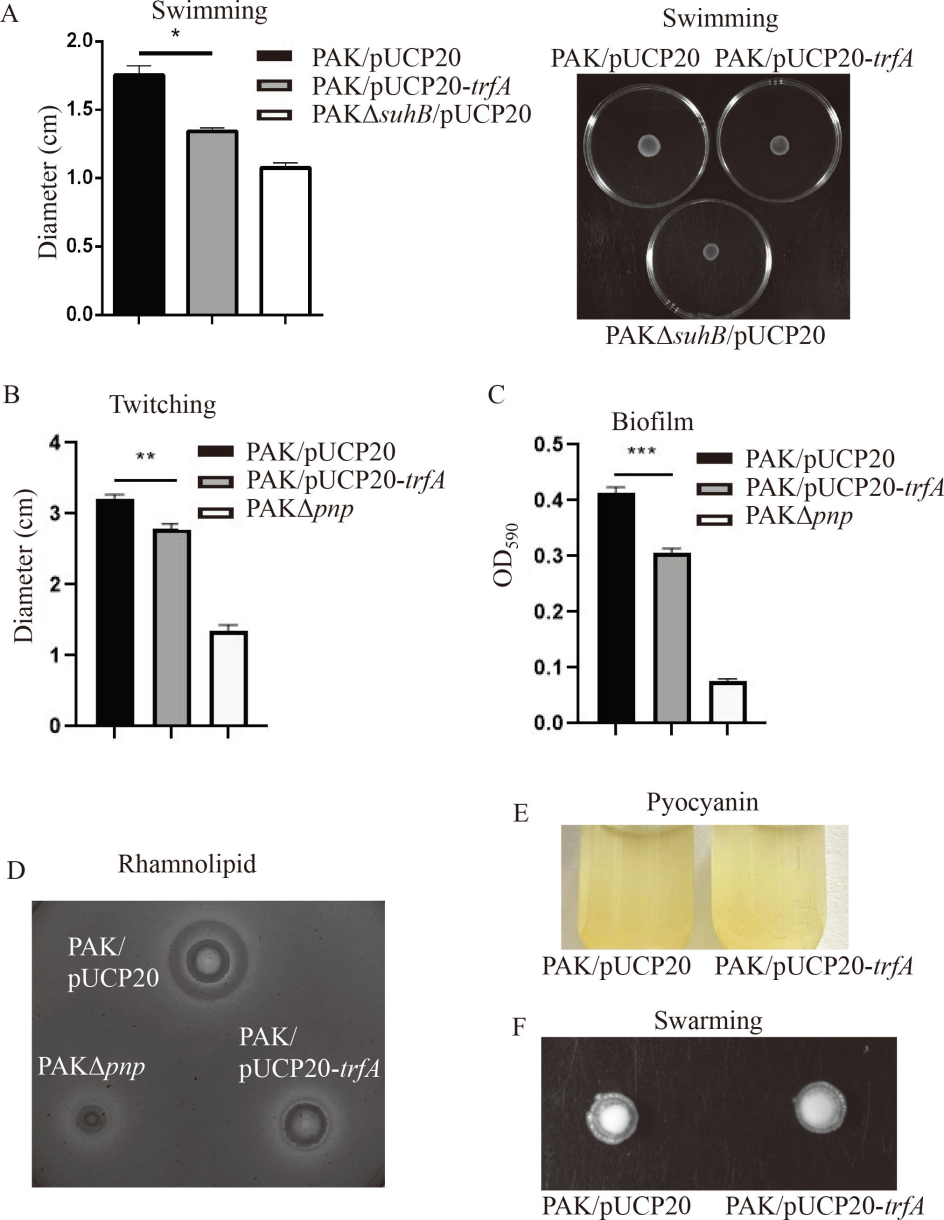
The effect of TrfA on other phenotypes. (**A and B**) Diameters of the swimming zone (A, left panel for diameters and right panel for the swimming images) and twitching zone (**B**) for PAK/pUCP20, PAK/pUCP20-*trfA,* and PAKΔ*suhB*/pUCP20 (as control for swimming motility in A) or PAKΔ*pnp* (as control for twitching motility in B). (**C**) Biofilm formation. Overnight cultures of PAK/pUCP20, PAK/pUCP20-*trfA,* and PAKΔ*pnp* (as control for biofilm production) were diluted 50-fold in LB and incubated in each well of a 96-well plate for 24 h at 37°C. The biofilm was stained with 0.25% crystal violet for 15 min, washed with water three times, dissolved in 200 µL of the eluate, and subjected to measurement at OD_590_. (**D**) Rhamnolipid production was examined on the plate. One microliter culture of the PAK/pUCP20, PAK/pUCP20-*trfA,* and PAKΔ*pnp* (as control for rhamnolipid production) strains was plotted onto the plate and incubated at 37°C for 24 h and then further at room temperature for 72 h. The halo ring around the bacterial colony indicates the production of rhamnolipids. (**E**) Pyocyanin production. (**F**) Swarming motility of PAK/pUCP20 and PAK/pUCP20-*trfA*. *P < 0.05, **P < 0.01, ***P < 0.001 compared with PAK/pUCP20 by Student’s *t* test.

To determine the global effect of TrfA in *P. aeruginosa*, we examined the motility, biofilm formation ability, and production of rhamnolipid and pyocyanin in the PAK/pUCP20-*trfA* and PAK/pUCP20 strains with PAKΔ*suhB* or PAKΔ*pnp* as control which had been demonstrated to affect the swimming motility or twitching motility, biofilm forming, and rhamnolipid production (24, 25). As shown in Fig. 7, the expression of *trfA* in PAK resulted in decreased swimming, twitching, biofilm forming ability, and rhamnolipid production (Fig. 7A through D), whereas it had no obvious influence on swarming and pyocyanin production compared to PAK/pUCP20 (Fig. 7E and F).

## DISCUSSION

Plasmid RK2, initially identified in *P. aeruginosa*, has been extensively studied for understanding plasmid DNA replication, stable inheritance, conjugative transfer, and the promiscuous nature of the IncP-1 plasmid (26). TrfA, the plasmid replication initiation protein, is required for RK2 replication in a broad range of hosts (27). In this study, we found that TrfA represses the expression of T3SS genes in *P. aeruginosa*. In addition to modulating the expression of PA5530 and cAMP levels, TrfA directly binds to the promoter of the *exsCEBA* operon to suppress the T3SS expression. It is well known that host chromosome-encoded protein factors control plasmid replication (28). To the best of our knowledge, this is the first report for a plasmid replication initiation protein modulating the expression of host chromosomal genes.

PA5530, encoding a dicarboxylate transporter, is responsible for the uptake of extracellular α-ketoglutarate and essential for the growth of *P. aeruginosa* on α-ketoglutarate and glutarate (two C5-dicarboxylates) (29). C5-dicarboxylates can induce the expression of the PA5530 gene via enhancer binding protein MifR and alternative sigma factor RpoN (29). In the present study, we found that TrfA activates the expression of PA5530. Overproduction of PA5530 decreased the intracellular cAMP levels and reduced the expression of T3SS in *P. aeruginosa* PAK. When expressing in *E. coli* BL21, TrfA has no influence on the promoter activity of P_PA5530_*-lacZ* (data not shown), indicating a possible indirect regulation of PA5530 mediated by TrfA. However, the molecular mechanism underlying the TrfA-mediated upregulation of PA5530 remains elusive and warrants further studies.

The production of TrfA resulted in decreased intracellular cAMP in *P. aeruginosa*. Based on the facts that the expression of *cyaB* and ATP levels were decreased by TrfA and ectopic expression of *cyaB* increased the cAMP level in PAK/pUCP20-*trfA*, but not to the levels of PAK/pUCP20, it is possible that both decreased ATP levels and CyaB amounts contribute to the reduced cAMP levels in the PAK/pUCP20-*trfA* strain. In addition to the T3SS, the expression of *trfA* also reduced swimming, twitching motility, biofilm formation, and rhamnolipid production in *P. aeruginosa*. Since twitching motility was regulated by the cAMP-Vfr signaling system (13), we postulate that TrfA might exert its effect on it via the cAMP-Vfr pathway. Of note, while these phenotypes were decreased by the expression of *trfA*, the expression of the phenotype-related genes was not altered by TrfA according to the RNAseq analysis. We infer that this might be due to the significance standard for differentially expressed genes used in the RNAseq analysis. For the RNAseq, differentially expressed genes were identified with a fold change larger than 2 and q-value no more than 0.05 as cutoff values. Several genes related to the altered phenotypes were excluded from the analysis with a fold change greater than 2 but a q value more than 0.05 (volcano plot in Fig. S4, and full RNAseq data in Table S2). In addition, the transcriptome results revealed decreased transcription of H1-T6SS in PAK/pUCP20-*trfA* and real-time qPCR assay further confirmed the reduced expression of H1-T6SS (Fig. S5). And also, *hcnB* gene, encoding a hydrogen cyanide synthase HcnB, is also decreased in the RNAseq data (Table S1). Hydrogen cyanide biosynthetic genes have been reported to be transcriptionally controlled by quorum sensing regulators LasR and RhlR in *P. aeruginosa* (30). Since Vfr, the cAMP receptor protein, controls quorum sensing in *P. aeruginosa* (31), it is possible that TrfA regulates *hcnB* through the cAMP-Vfr-quorum sensing pathway. Of note, TrfA has been demonstrated to bind to a conserved 17 bp sequence in the *oriV* of the RK2 plasmid (32). However, the conserved sequence was not found in the promoter region of P*_exsC_*. Therefore, although no conserved 17 bp sequence exists in the promoter region of *hcnABC* operon, the direct regulation of *hcnB* by TrfA cannot be excluded at present. Currently, we are making efforts to explore the molecular mechanism of TrfA-mediated regulation of the H1-T6SS in *P. aeruginosa*.

RK2 was originally identified to confer antibiotic resistance to *P. aeruginosa* isolated from burn patients (15). Here, we show TrfA, the replication initiation protein of RK2, suppresses production of the virulence factors of *P. aeruginosa*. Therefore, antibiotic resistance acquisition from RK2 caused a decrease in virulence factor production. Interestingly, this is not the first case of a negative correlation between virulence factor production and resistance to antibiotics. *P. aeruginosa* respiratory isolates lacking elastase production are more resistant to ceftazidime and piperacillin, and nonpyocyanin-producing strains are less susceptible to amikacin, tobramycin, ceftazidime, ciprofloxacin, and ofloxacin (33). It has been demonstrated that overexpression of efflux pumps leads to an increase in antibiotic resistance but a reduction in the production of diverse virulence factors in *P. aeruginosa* (34, 35). Although TrfA-encoding plasmids have been found in both clinical settings and environments from time to time (36–39), much attention was paid to the antibiotic resistance conferred by them, without consideration of their influence on the host genomic gene expression. Interestingly, an IncP1 plasmid p27003_KPC, containing *trfA* isolated from a clinical *Klebsiella pneumoniae* strain (36), reduced the expression and secretion of T3SS in *P. aeruginosa* (Fig. S6). Much more investigations are needed to elucidate the clinical relevance other than antibiotic resistance conferred by TrfA-encoding and non-encoding plasmids in the future.

In addition to RK2, TrfA was also encoded by all other IncP-1 plasmids, which were discovered in a diversity of bacterial hosts (40). According to the analysis of the nucleotide blast on the NCBI (https://blast.ncbi.nlm.nih.gov/), *trfA* was identified in the genome of several bacteria, including *E. coli* S17-1, *E. coli* strain ST18 and MFDpir, *Caulobacter sp*. “ethensis” strain CETH2.0, *Acinetobacter sp.* FDAARGOS_493, and *Burkholderia glumae* strain PW30RS. Evolutionarily, whether TrfA originated from chromosome or plasmid is not known. In addition, whether TrfA, encoded in extrachromosomal plasmids or chromosomes, plays a role in the chromosomal gene expression of other bacteria remains unknown and warrants further investigation.

Taken together, our study showed that the plasmid replication initiator TrfA decreases intracellular cAMP and suppresses the expression of T3SS. TrfA also binds to P*_exsC_* and inhibits *exsA* transcription to repress the T3SS in *P. aeruginosa*. Further studies are required to understand the detailed regulatory mechanisms of TrfA and its regulatory roles in other bacterial species.

## MATERIALS AND METHODS

### Bacterial strains and plasmids

The bacterial strains, plasmids, and primers used in this study are listed in Tables S3 and S4. All strains were grown in LB medium (10 g/L tryptone, 5 g/L NaCl, and 5 g/L yeast extract) or on L-agar plates (LB medium supplemented with 15 g/liter agar) at 37°C. When needed, antibiotics were used at the following concentrations (μg/mL): for *E. coli*, ampicillin 100, gentamicin 10, kanamycin 25, tetracycline 10; for *P. aeruginosa*, carbenicillin 150, gentamicin 100, tetracycline 50. When needed, IPTG (isopropyl β-D-1-thiogalactopyranoside) was added to the culture medium at a final concentration of 1 mM. The primers used to make constructs and for real-time qPCR are listed in Table S4.

 For the expression of the *trfA* gene, the open reading frame of *trfA* and its putative SD sequence were PCR-amplified with pDN19 plasmid DNA as the template. The PCR product was digested and then ligated into the *Bam*HI-*Eco*RI sites of pUCP20, resulting in pUCP20-*trfA*. pUCP20-*TcR*, pUCP20-*traJ*, pUCP20-PA1137, pUCP20-PA1523, pUCP20-PA5530, pUCP20-*trfA*-PA5530, pUCP20-PA5530-*cyaB*, pUCP20-*trfA-cyaB,* and pET28a-*trfA*-His were constructed with similar manipulations. For pUCP20-*trfA*-PA5530, pUCP20-PA5530-*cyaB* and pUCP20-*trfA-cyaB*, two genes have separate ribosome-binding sites and were driven by the same promoter P*_lac_* on pUCP20. The PA5530 deletion construct was made by cloning 1,009 bp upstream and 998 bp downstream fragments of the PA5530 gene into the *Eco*RI-*Hin*dIII sites of plasmid pEX18Tc (41). To generate the P*_cyaA_-lacZ* construct, the putative promoter region of *cyaA* was amplified from PAK genomic DNA and cloned into the *Eco*RI-*Kpn*I sites of pDN19lacZΩ. P*_cyaB_-lacZ* was constructed with similar manipulations.

### Western blot analysis

Overnight cultures of bacteria were diluted 50-fold into fresh LB medium with or without 5 mM EGTA and cultured to an OD_600_ of 1.0. Supernatant and/or pellet samples were separated by centrifugation at 13,000 × *g* for 2 min. Protein samples from equivalent numbers of bacterial cells were separated on 12% sodium dodecyl sulfate-polyacrylamide gel (SDS-PAGE), transferred onto a PVDF (polyvinylidene difluoride) membrane (Millipore), and hybridized with a rabbit polyclonal antibody against ExoS or mouse monoclonal antibody against RpoA (BioLegend). The RNA polymerase alpha subunit RpoA was used as a loading control. Signals were detected using the ECL Plus kit (Millipore).

### RNA isolation, RNAseq, and real-time qPCR

RNA isolation and real-time qPCR were performed following the manufacturer’s instructions with minor modifications. Overnight cultures of *P. aeruginosa* strains were diluted 50-fold into fresh LB medium containing 0 or 5 mM EGTA and grown to an OD_600_ of 1.0 at 37°C. Total RNA was extracted using an RNAprep Pure cell/Bacteria Kit (Tiangen Biotech, Beijing, China) and then reverse-transcribed into cDNA using random primers and PrimeScript Reverse Transcriptase (TaKaRa, Dalian, China). Real-time qPCR was performed in a CFX connect real-time machine (Bio-Rad, Hercules, CA, USA) with the indicated primers (Table S4) and SYBR Premix ExTaq II (TaKaRa, Dalian, China). *rpsL*, a 30S ribosomal protein-encoding gene, was utilized as an internal control. For RNAseq, total RNAs were isolated from bacteria grown in LB medium without EGTA induction as above and then sent to GENEWIZ Life Sciences (Suzhou, China), where library construction, sequencing, and analysis were carried out as described in our previous study (42). The raw reads are 26,339,614 and 29,336,200 for *PAK/pUCP20-trfA* and PAK/pUCP20, respectively. The raw RNA sequencing data have been deposited in the NCBI database under accession code PRJNA948711.

### Mouse acute pneumonia model

A mouse acute pneumonia model was constructed following a previous description (22). Briefly, an overnight culture of *P. aeruginosa* was diluted 50-fold into fresh LB medium and grown to an OD_600_ of approximately 1.0 at 37°C. Bacteria were collected, washed twice with phosphate-buffered saline (PBS), and adjusted to 5 × 10^8^ CFU/mL in PBS. Each female BALB/c mouse, at the age of 6–8 weeks, was anesthetized with an intraperitoneal injection of 100 µL 7.5% chloral hydrate. Forty microliters of bacterial suspension was intranasally inoculated into each mouse, leading to 2 × 10^7^ CFU in each mouse. At 12 h post-infection, the mice were sacrificed via CO_2_ inhalation, and the lungs were isolated and homogenized with 1% proteose peptone (Solarbio, Beijing, China). CFU counts were carried out by plating assay.

### Expression and purification of proteins from *E. coli*

The TrfA coding sequence was amplified by PCR using pDN19 plasmid DNA as the template with specific primers (Table S4). The gene was inserted into the *Nco*I-*Xho*I sites of the pET28a vector, leading to TrfA translational fusion with the His tag at the C-terminus. *E. coli* strain BL21 (DE3) harboring pET28a-*trfA* was cultured at 37°C to an OD_600_ of 0.4 to 0.6, and the expression of His-tagged TrfA was induced with 1 mM IPTG (Solarbio, Beijing, China) for 16 h at 16°C. Bacterial cells were collected from 200 mL cultures by centrifugation at 8,000 × *g* for 10 min, resuspended in 10 mL lysis buffer (46.6 mM Na_2_HPO_4_, 3.4 mM NaH_2_PO_4_, 0.3 M NaCl, 20 mM imidazole, pH 8.0), and lysed by sonication on ice. After centrifugation at 12,000 × *g* for 10 min at 4°C, proteins were purified from the supernatant with Ni-NTA agarose (Qiagen) and incubated at 4°C for 1 h. After that, the lysate-resin mixture was loaded into an empty column and washed twice with lysis buffer containing 20 mM imidazole. The TrfA-His protein was eluted with 1 mL of lysis buffer containing 200 mM imidazole.

### Electrophoretic mobility shift assay (EMSA)

EMSA was carried out following a previous description with minor modifications (43). The DNA fragments corresponding to the promoter region of P*_exsC_*, *oriV* of pDN19, and upstream region of *algD* were amplified using PCR with specific primers (Table S4). 5′-end labeled 6-carboxyfluorescein (6-FAM) primers (Table S4) were used to amplify the respective FAM-labeled DNA fragments. Ten nanograms of 6-FAM labeled DNA probes was incubated with 0 or 0.9 µM purified recombinant TrfA protein in a 20 µL reaction containing 20 mM Tris-HCl (pH 7.4), 100 mM KCl, 7 mM MgCl_2_, 1 mM EDTA, 1 mM DTT, and 5% glycerol on ice for 30 min. Competitive reaction mixtures were prepared with a 50-fold excess of unlabeled DNA competitors. Samples were loaded onto 12% native polyacrylamide gel that had been prerun at 100 V for 1 h in 0.5 × TBE buffer (44.5 mM Tris base, 44.5 mM boric acid, 1 mM EDTA, pH 8.0) and electrophoresed on ice at 10 mA for 1 h and 45 min. Afterward, the fluorescently labeled bands were visualized with an Amersham Typhoon Scanner.

### Motility, rhamnolipid, pyocyanin, and biofilm formation assays

Three kinds of media were utilized to examine swimming (5 g/L NaCl, 10 g/L tryptone, and 0.3% agarose), swarming (62 mM K_3_PO_4_, 2 mM MgSO_4_, 10 mM FeSO_4_, 0.4% glucose, 0.1% casein hydrolysate, and 0.35% agar), and twitching (LB medium containing 1% agar) motility as previously described with minor modifications (44). *P. aeruginosa* strains were cultured overnight, and 1 µL of the culture was spotted onto the surface (swimming and swarming) or stabbed into the agar plates (twitching). After absorption of the inoculum, the plates were incubated at 30°C for 20 h (swimming motility) and 37°C for 20 h (swarming) or 18 h (twitching motility). Diameters were measured for swimming and twitching motility, while images of the swarming plates were taken using a ChemiDocTM XRS + imager (Bio-Rad, software version 4.0.0.49710). Bacteria were grown overnight at 37°C in LB medium, and then pyocyanin production was assayed via observation and imaging of culture color. Quantifications of biofilm were determined as described previously (45). Determination for rhamnolipid production was carried out as previously described with modifications (46). Briefly, 1 µL overnight bacterial culture (in LB medium) was dropped onto the rhamnolipid assay agar plate (0.9 g Na_2_HPO_4_, 0.7 g KH_2_PO_4_, 0.71 g MgSO_4_·7H_2_O, 2 g NaNO_3_, 0.001 g CaCl_2_·2H_2_O, 0.001 g FeSO_4_·7H_2_O, 11 g glucose per liter, pH 7.4; supplemented with 0.5% casein (m/v), 1.6% agar (m/v), 0.02% (m/v) cetyltrimethylammonium bromide, and 0.01% (m/v) methylene blue), and cultured at 37°C for 24 h. After that, the plate was kept for more than 72 h at room temperature. Rhamnolipid production was assayed by observing violet halos around the bacterial colonies.

### Other methods

PA5530 and *exsD* gene deletion in the PAK strain were carried out by homologous recombination, as previously described (41). The β-galactosidase activity was measured to examine the promoter transcriptional activity, as described previously (47). The minimum inhibitory concentration (MIC) of tetracycline was tested by a twofold serial dilution method (48).

## SUPPLEMENTAL MATERIAL

Supplemental figures mbio.02784-25-s0001.pdf Fig. S1-S6.

Table S1 mbio.02784-25-s0002.xls Altered genes in PAK/pUCP20-*trfA* compared to PAK/pUCP20.

Table S2 mbio.02784-25-s0003.xls All RNAseq data in PAK/pUCP20-*trfA* compared to PAK/pUCP20.

Table S3 mbio.02784-25-s0004.docx Bacterial strains and plasmids used in this study.

Table S4 mbio.02784-25-s0005.doc Primers used in this study.

ASM does not own the copyrights to Supplemental Material that may be linked to, or accessed through, an article. The authors have granted ASM a non-exclusive, world-wide license to publish the Supplemental Material files. Please contact the corresponding author directly for reuse.
