# Supplementary material for: Plasmid replication initiator protein TrfA represses the host type III secretion system in Pseudomonas aeruginosa
Source: mBio. 2025 Nov 5;16(12):e02784-25. doi: 10.1128/mbio.02784-25 (PMC12691604; doi:10.1128/mbio.02784-25)
Supplement: Supplemental figures — Fig. S1-S6. [file mbio.02784-25-s0001.pdf]

Fig. S1

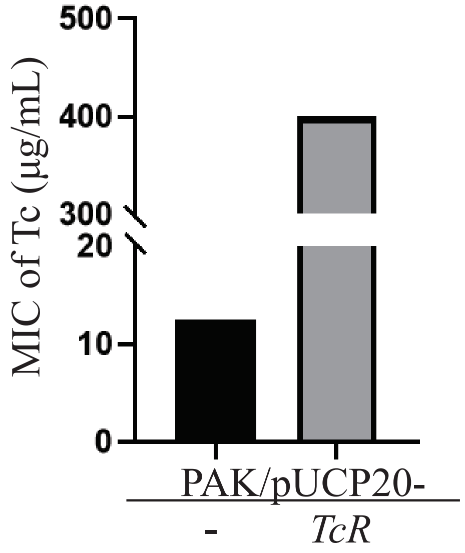

Fig. S1 MIC of tetracycline for PAK/pUCP20 and PAK/pUCP20-*TcR*

Fig. S2

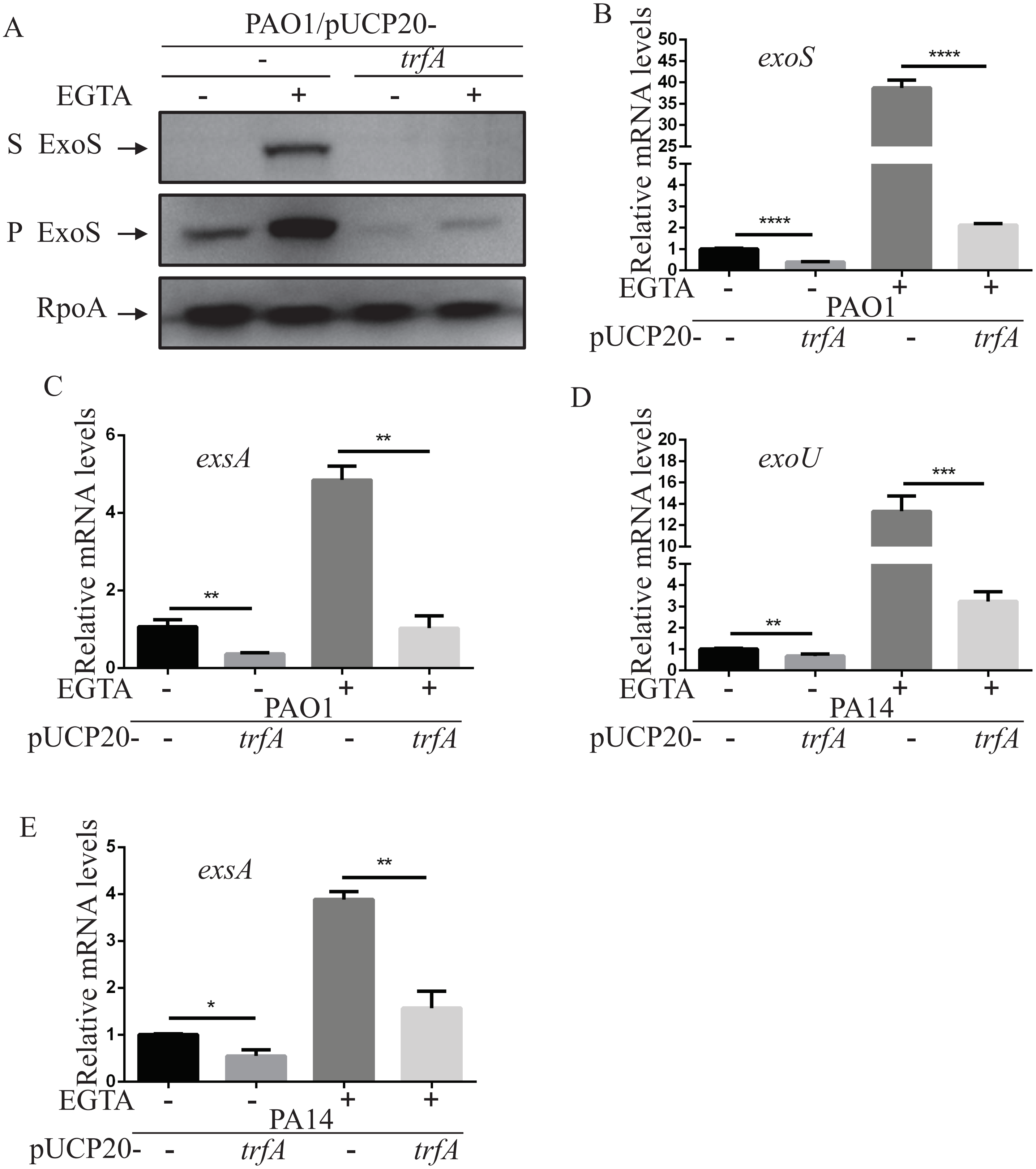

Fig. S2 TrfA represses T3SS in PAO1 and PA14 strains. (A) Expression and secretion of ExoS in the indicated strains. Bacterial cells were grown to an OD_{600} of 1.0 in LB with 0 (-) or 5 mM (+) EGTA. Proteins in supernatants (S) and pellets (P) from equivalent bacterial cells were separated by 12% SDS-PAGE gels and probed with anti-ExoS antibody or anti-RpoA antibody.(B-E) Relative mRNA levels of *exoS*, *exsA*, and *exoU* in indicated strains. **P* < 0.05, ***P* < 0.01, ***P* < 0.001, *****P*<0.0001, by Student's *t* test.

Fig. S3

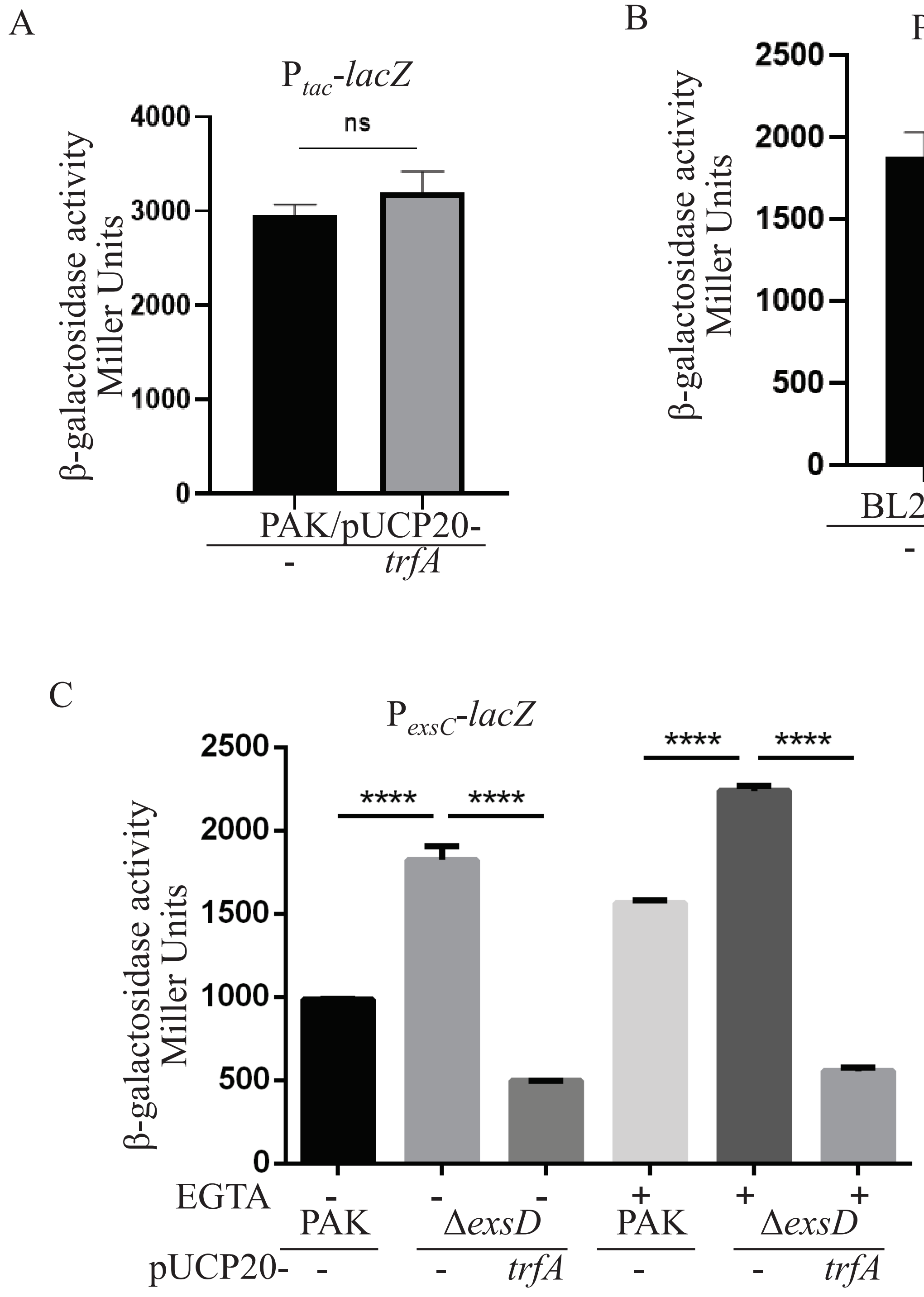

Fig. S3 β-galactosidase activity assay. The indicated bacterial strains containing the P_{tac}-*lacZ* (A and B) or P_{exsC}-*lacZ* (C) transcriptional reporter plasmid were grown to an OD_{600} of 1.0 in LB [A, B and C(EGTA-)] or LB with 5 mM EGTA (EGTA+ in C) and subjected to β-galactosidase assays. Each assay was performed in triplicate, and the error bars indicate standard deviations. ns, not significant, ****P<0.0001, by Student's *t* test.

Fig. S4

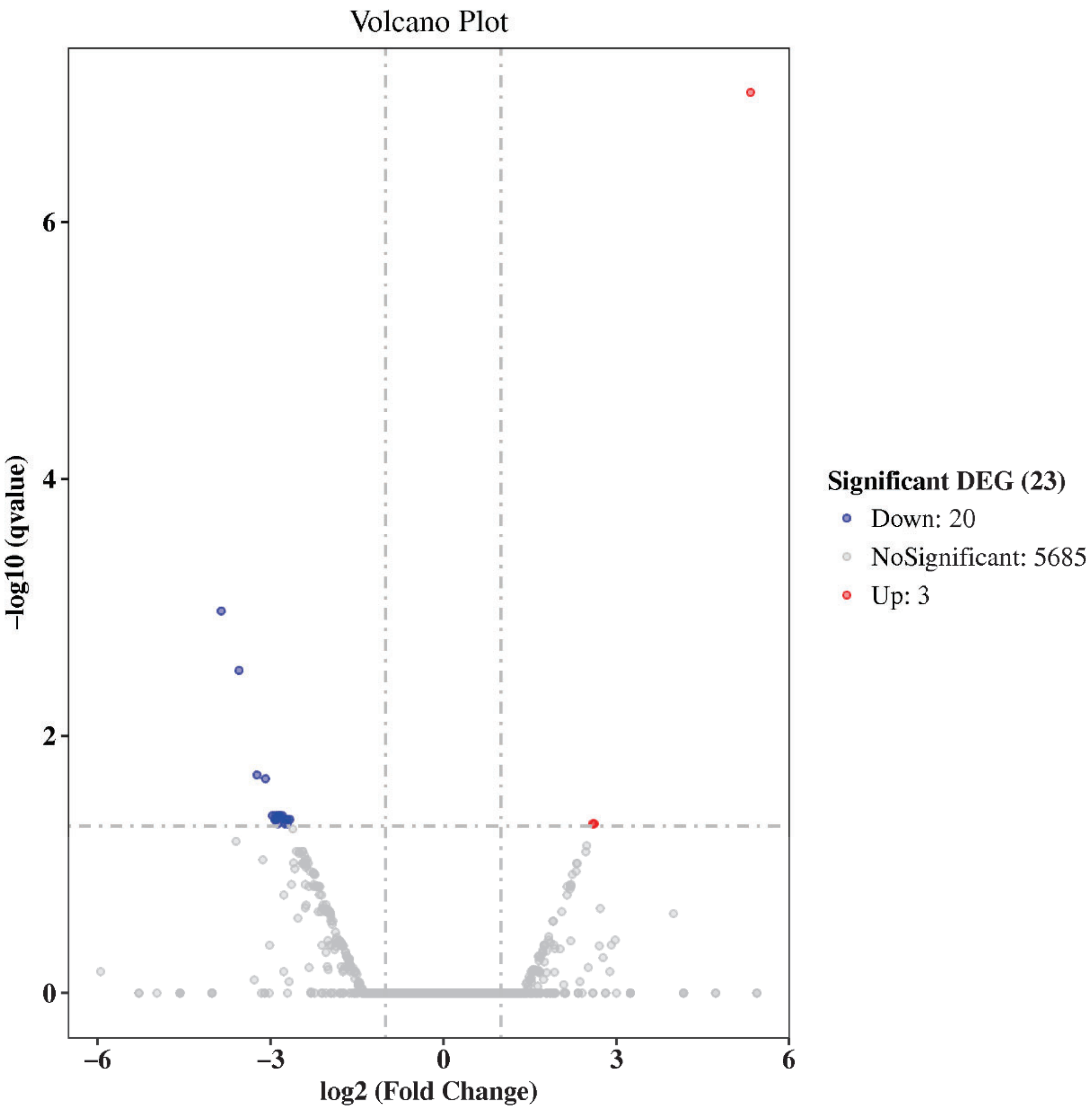

Fig. S4 Volcano plot displaying the differences in transcriptional profiles between PAK/pUCP20-*trfA* and PAK/pUCP20. The X and y axes represent, respectively, the $\log_2$ (fold chage) and $-\log_{10}$ (qvalue) of all genes. The red dot represents upregulation, the blue dot represents downregulation, and the grey dot represents no significant change.

Fig. S5

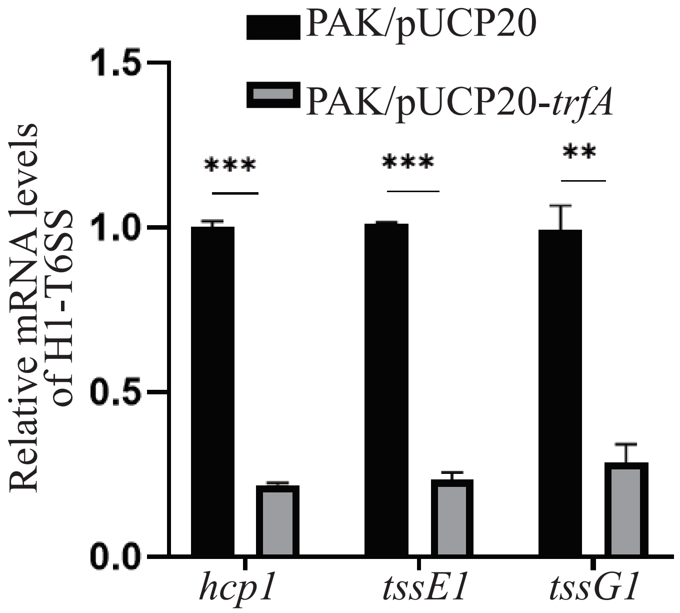

Fig. S5 Relative mRNA levels of *hcp1*, *tssE1*, and *tssG1* in PAK/pUCP20 and PAK/pUCP20-*trfA* strains. Total RNA was isolated under T3SS noninducing conditions, and the relative mRNA levels were determined by real-time qPCR using *rpsL* as the internal control. $^{**}P < 0.01$, $^{***}P < 0.001$ compared with PAK/pUCP20 by Student's *t* test.

Fig. S6

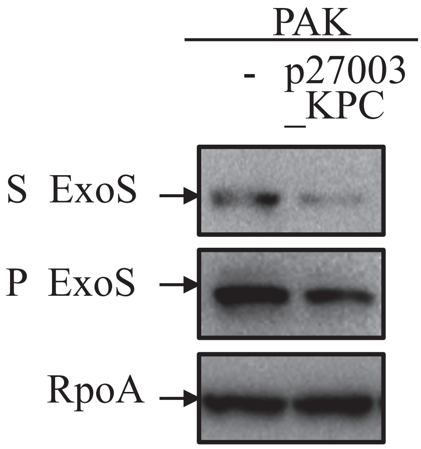

Fig. S6 Clinical plasmid containing *trfA* represses ExoS expression and secretion in PAK strain. Expression and secretion of ExoS in the indicated strains. Bacterial cells were grown to an OD_{600} of 1.0 in LB with 5 mM EGTA. Proteins in supernatants (S) and pellets (P) from equivalent bacterial cells were separated by 12% SDS-PAGE gels and probed with anti-ExoS antibody or anti-RpoA antibody.
