# Supplementary material for: Plasmid replication initiator protein TrfA represses the host type III secretion system in Pseudomonas aeruginosa
Source: mBio. 2025 Nov 5;16(12):e02784-25. doi: 10.1128/mbio.02784-25 (PMC12691604; doi:10.1128/mbio.02784-25)
Supplement: Table S3 — Bacterial strains and plasmids used in this study. [file mbio.02784-25-s0004.docx]

**Table S3.** Bacterial strains and plasmids used in this study.

| Strains or plasmids | Description | Source or reference |
| --- | --- | --- |
| **strains** |  |  |
| DH5α | F^̶^ ϕ 80d*lacZ*∆M15 *endA1 recA1 hsdR17*(r_K_^̶^ m_K_^+^) *supE44 thi-1 relA1* ∆(*lacZYA-argF*)*U169 gyrA96 deoR* | TransGen |
| S17-1 | RP4-2 Tc::Mu Km::Tn*7* Tp^r^ Sm^r^ Pro Res^̶^ Mod^+^ | Stratagene |
| BL21 (DE3) | F^-^ *ompT* *hsdSB (rB-, mB-) gal dcm* (DE3) | invitrogen |
| PAK | Wild type *P. aeruginosa* strain | David Bradley |
| PAKΔ*exsA* | PAK with *exsA* gene deleted | [1] |
| PAKΔPA5530 | PAK with PA5530 gene deleted | This study |
| PAKΔ*exsD* | PAK with *exsD* gene deleted | This study |
| PA14 | Wild type *P. aeruginosa* strain | [2] |
| PAO1 | Wild type *P. aeruginosa* strain | [3] |
|  |  |  |
| **Plasmids** |  |  |
| pUCP20 | Shuttle vector between *E. coli* and *P. aeruginosa*; Amp^r^ | [4] |
| pDN19 | Shuttle vector between *E. coli* and *P. aeruginosa*; Tc^r^ | [5] |
| pUCP20-*TcR* | Tcr coding gene from pDN19 in pUCP20; Amp^r^ | This study |
| pUCP20-*trfA* | *trfA* gene from pDN19 in pUCP20; Amp^r^ | This study |
| pUCP20-*traJ* | *traJ* gene from pDN19 in pUCP20; Amp^r^ | This study |
| pUCP20-PA1137 | PA1137 gene from PAK in pUCP20; Amp^r^ | This study |
| pUCP20-PA1523 | PA1523 gene from PAK in pUCP20; Amp^r^ | This study |
| pUCP20-PA5530 | PA5530 gene from PAK in pUCP20; Amp^r^ | This study |
| pUCP20-*trfA*-PA5530 | PA5530 gene from PAK in pUCP20-*trfA*; Amp^r^ | This study |
| pUCP20-*trfA*-*cyaB* | *cyaB* gene from PAK in pUCP20-*trfA*; Amp^r^ | This study |
| pUCP20-PA5530-*cyaB* | PA5530 and *cyaB* gene from PAK in pUCP20; Amp^r^ | This study |
| pMMB67EH-*exsA* | *exsA* gene from PAK in pMMB67EH; Amp^r^ | [1] |
| pEX18Tc | Gene knockout vector; Tc^r^ | [6] |
| pEX18Tc-PA5530 | PA5530 gene deletion on pEX18Tc; Tc^r^ | This study |
| P*_exsC_*-*lacZ* | *exsC* promoter fused to promoterless *lac*Z on pDN19*lac*ZΩ; Sp^r^, Sm^r^, Tc^r^ | [7] |
| P*_exsA_*-*lacZ* | *exsA* own promoter fused to promoterless *lac*Z on pDN19*lac*ZΩ; Sp^r^, Sm^r^, Tc^r^ | [7] |
| P*_lacP1_*-*lacZ* | *lac*P1 promoter of *E. coli* fused to promoterless *lacZ* on pDN19*lacZ*Ω; Sp^r^, Sm^r^, Tc^r^ | [8] |
| P*_tac_*-*lacZ* | *tac* promoter fused to promoterless *lac*Z on pDN19*lac*ZΩ; Sp^r^, Sm^r^, Tc^r^ | This study |
| P*_cyaA_*-*lacZ* | *cyaA* promoter fused to promoterless *lac*Z on pDN19*lac*ZΩ; Sp^r^, Sm^r^, Tc^r^ | This study |
| P*_cyaB_*-*lacZ* | *cyaB* promoter fused to promoterless *lac*Z on pDN19*lac*ZΩ; Sp^r^, Sm^r^, Tc^r^ | This study |
| pET28a | expression vector, Kan^r^ | Novagen |
| pET28a-*trfA* | *trfA* gene from pDN19 cloned into pET28a expression vector, Kan^r^ | This study |
| p27003_KPC | IncP1 plasmid from clinical isolate | [9] |

1. Yin, L., et al., *MvaT binds to the P(exsC) promoter to repress the type III secretion system in Pseudomonas aeruginosa.* Front Cell Infect Microbiol, 2023. **13**: p. 1267748.

2. Liberati, N.T., et al., *An ordered, nonredundant library of Pseudomonas aeruginosa strain PA14 transposon insertion mutants.* Proc Natl Acad Sci U S A, 2006. **103**(8): p. 2833-8.

3. Wang, D., et al., *RplI interacts with 5' UTR of exsA to repress its translation and type III secretion system in Pseudomonas aeruginosa.* PLoS Pathog, 2022. **18**(1): p. e1010170.

4. West, S.E., et al., *Construction of improved Escherichia-Pseudomonas shuttle vectors derived from pUC18/19 and sequence of the region required for their replication in Pseudomonas aeruginosa.* Gene, 1994. **148**(1): p. 81-6.

5. Nunn, D., S. Bergman, and S. Lory, *Products of three accessory genes, pilB, pilC, and pilD, are required for biogenesis of Pseudomonas aeruginosa pili.* J Bacteriol, 1990. **172**(6): p. 2911-9.

6. Schweizer, H.P., *Allelic exchange in Pseudomonas aeruginosa using novel ColE1-type vectors and a family of cassettes containing a portable oriT and the counter-selectable Bacillus subtilis sacB marker.* Mol Microbiol, 1992. **6**(9): p. 1195-204.

7. Deng, X., et al., *Fis Regulates Type III Secretion System by Influencing the Transcription of exsA in Pseudomonas aeruginosa Strain PA14.* Front Microbiol, 2017. **8**: p. 669.

8. Jin, Y., et al., *NrtR Regulates the Type III Secretion System Through cAMP/Vfr Pathway in Pseudomonas aeruginosa.* Front Microbiol, 2019. **10**: p. 85.

9. Yan, R., et al., *A Sequence Type 23 Hypervirulent Klebsiella pneumoniae Strain Presenting Carbapenem Resistance by Acquiring an IncP1 bla(KPC-2) Plasmid.* Front Cell Infect Microbiol, 2021. **11**: p. 641830.
