# Supplementary material for: Plasmid replication initiator protein TrfA represses the host type III secretion system in Pseudomonas aeruginosa
Source: mBio. 2025 Nov 5;16(12):e02784-25. doi: 10.1128/mbio.02784-25 (PMC12691604; doi:10.1128/mbio.02784-25)
Supplement: Table S4 — Primers used in this study. [file mbio.02784-25-s0005.doc]

**Table S4**. Primers used in this study.

| Primer^a^ | Sequence 5’-3’^c^ | Use |
| --- | --- | --- |
| *TcR*-F | CGGAATTCGGCTGGTCCGGAGGCCAGAC | pUCP20-*TcR* |
| *TcR*-R | CGGGATCCTCAGCGATCGGCTCGTTGCCC |
| *traJ*-F | CGGAATTCTAACAGATGAGGGCAAGCGG | pUCP20-*traJ* |
| *traJ*-R | CGGGATCCTCATGGCTCTGCCCTCGGGCGG |
| *trfA*-F | CGGAATTCCAATCCCGCAAGGAGGGTGA | pUCP20-*trfA* |
| *trfA*-R | CGGGATCCCTAGCGTTTGCAATGCACCA |
| *trfA*-His-F | CATGCCATGGATCGGACGTTTGACCGGAA | pET28a-*trfA*-His |
| *trfA*-His-R | CCGCTCGAGGCGTTTGCAATGCACCAGGTCA |
| PA5530-F | GGAATTCTGATCAGCACATCCAAGAC | pUCP20-PA5530 |
| PA5530-R | CCCAAGCTTTCAATCGGTCGTGATCTTC |
| PA1137-F | GGAATTCGCGAACCAGGTCCGAACC | pUCP20-PA1137 |
| PA1137-R | CCCAAGCTTTCAGGGAGCTGATGTGAGG |
| PA1523-F | GGAATTCCTTCCTGGAGCTGCACGCTC | pUCP20-PA1523 |
| PA1523-R | CCCAAGCTTTCAGGCCGGTTCGACCGCG |
| PA5530-F1 | CGCGGATCCCAACGAAAAGAGGAAACACGATGG | pUCP20-*trfA*-PA5530 |
| PA5530-R1 | CCCAAGCTTTCAATCGGTCGTGATCTTCGAGT |
| *cyaB*-F1 | CGCGGATCCCGAGCCGGTCCGTCGC | pUCP20-*trfA*-*cyaB* |
| *cyaB*-R1 | CCCAAGCTTTTAGAGGATGACCTTGTCGCGC |
| PA5530-F2 | CCGGAATTCCAACGAAAAGAGGAAACACGATGG | pUCP20-PA5530-*cyaB* |
| PA5530-R2 | CGCGGATCCTCAATCGGTCGTGATCTTCGAGT |
| PA5530-UF | GGAATTCCGACTTCCTCTACCCGGTG | pEX18-PA5530 |
| PA5530-UR | GGGGTACCCGTGTTTCCTCTTTTCGTTGTTG |
| PA5530-DF | GGGGTACCTTGAGCCTCGTGCTCACGG |
| PA5530-DR | CCCAAGCTTGACGCCGCCGAAGATCGAG |
| *exsD*-UF | CCTGCAGGTCGACTCTAGAGGTATTCATGCCTCTCCGCTAGGC | pEX18-*exsD* |
| *exsD*-UR | ATCCATGATTCCTCGGTAGGGCTCTCTGCCTTGGCTTCCTCACTAC |
| *exsD*-DF | GCCCTACCGAGGAATCATGGAT |
| *exsD*-DR | ATTCGAGCTCGGTACCCGGGAAGATCTCCACGCTGAGGTCG |
| pDN19*ori*-F^b^ | CGGCGACGTGGAGCTGGCCA | For EMSA |
| pDN19*ori*-R | GGAGGGTTCGAGAAGGGGGG |
| *algD*-F | CAGGGGTGTCGGAGGGACGAACGGTA | For EMSA |
| *algD*-R | ACGGCTATTACTTCAGCGCCGAGCAATC |
| P*_exsC_*-F^b^ | CGCCTCCTAAAGCTCAGCGCATGC | For EMSA |
| P*_exsC_*-R | ATGAAGGACGTCCTGCAGCTCATCC |
| 19-tac-F | AATTCTTGACAATTAATCATCGGCTCGTATAATGG | pDN19-P*_tac_*-*lacZ* |
| 19-tac-R | GATCCCATTATACGAGCCGATGATTAATTGTCAAG |
| 19-*cyaA*-F | GGAATTCGCAGCGCATCCTCGCCAGC | pDN19-P*_cyaA_*-*lacZ* |
| 19-*cyaA*-R | GGGGTACCGGGCGTCCGGGCACAGGC |
| 19-*cyaB*-F | GGAATTCTCGCCGAGTTCTACCCCTAC | pDN19-P*_cyaB_*-*lacZ* |
| 19-*cyaB*-R | GGGGTACCGCGCTGGAGAGGATCCCTG |
| qPCR primer |  |  |
| q-*exoS-*F | GCATATTCAATCGCTTCAG | RT-qPCR of *exoS* |
| q-*exoS-*R | CCTCAATCTGTCCCAAAC |
| q-*exsA-*F | GCTATGTCGTAAGTACCA | RT-qPCR of *exsA* |
| q-*exsA-*R | GAAGCCTTGTAGAAACTG |
| q-*exsD*-F | AGAGGTGCGGCAGATTCTCC | RT-qPCR of *exsD* |
| q-*exsD*-R | GCAGCAGGACCCAATCGA |
| q-*rpsL-*F | CAAAACTGCCCGCAACGT | RT-qPCR of *rpsL* |
| q-*rpsL-*R | TTTCGGCGTGGTGGTGTAT |
| q-*cyaA*-F | CTTCAAGGAGCAGGTATTC | RT-qPCR of *cyaA* |
| q-*cyaA*-R | TTCGAGATGGCGATAGAC |
| q-*cyaB*-F | GCTCACCGTGTTCTTCTCCG | RT-qPCR of *cyaB* |
| q-*cyaB*-R | CCGAAGAACACCATGACGCA |
| q-PA5530-F | CTGGATCGGCTTCGAGATC | RT-qPCR of PA5530 |
| q-PA5530-R | GGGCGATGATGCCGATGCC |
| q-*hcp1*-F | AGGACCTGTCGTTCACCAA | RT-qPCR of *hcp1* |
| q-*hcp1*-R | ATAGTGCTTGCCGCTGGA |
| q-*tssE1*-F | CTGACCCTGAACCAGCTGAAG | RT-qPCR of *tssE1* |
| q-*tssE1*-R | GCAGCCCGTAGTTGAGCACC |
| q-*tssG1*-F | GACCAGTTCTTCTTCGGCCTC | RT-qPCR of *tssG1* |
| q-*tssG1*-R | GCAGGCGGTGGTGGAAGAC |
| q-PA1137-F | CTCGGACATTCCCGGCCTG | RT-qPCR of PA1137 |
| q-PA1137-R | CGTCGGCCACCGCGAACTG |
| q-PA1523-F | CTGACCCCGGTGAAGTTCGG | RT-qPCR of PA1523 |
| q-PA1523-R | CGTCGACCTGGAAGACCTCG |

a: F: forward; R, reverse; U, upstream of specific gene; D, downstream of specific gene; q, qPCR.

b: This primer was also labelled with 6-FAM and used to amplify the fragment for competitive EMSA.

c: The underlines are the sites of restriction enzymes and protective bases.
